# Neonatal Diabetes and Congenital Hyperinsulinism Caused by Mutations in *ABCC8*/SUR1 are Associated with Altered and Opposite Affinities for ATP and ADP

**DOI:** 10.3389/fendo.2015.00048

**Published:** 2015-04-15

**Authors:** David Ortiz, Joseph Bryan

**Affiliations:** ^1^Department of Medicinal Chemistry, University of Washington, Seattle, WA, USA; ^2^Pacific Northwest Diabetes Research Institute, Seattle, WA, USA

**Keywords:** neonatal diabetes, congenital hyperinsulinism, sulfonylurea receptor, K_ATP_ channels

## Abstract

ATP-sensitive K^+^ (K_ATP_) channels composed of potassium inward-rectifier type 6.2 and sulfonylurea receptor type 1 subunits (Kir6.2/SUR1)_4_ are expressed in various cells in the brain and endocrine pancreas where they couple metabolic status to membrane potential. In β-cells, increases in cytosolic [ATP/ADP]_c_ inhibit K_ATP_ channel activity, leading to membrane depolarization and exocytosis of insulin granules. Mutations in *ABCC8* (SUR1) or *KCNJ11* (Kir6.2) can result in gain or loss of channel activity and cause neonatal diabetes (ND) or congenital hyperinsulinism (CHI), respectively. SUR1 is reported to be a Mg^2+^-dependent ATPase. A prevailing model posits that ATP hydrolysis at SUR1 is required to stimulate openings of the pore. However, recent work shows nucleotide binding, without hydrolysis, is sufficient to switch SUR1 to stimulatory conformations. The actions of nucleotides, ATP and ADP, on ND (SUR1_E1506D_) and CHI (SUR1_E1506K_) mutants, without Kir6.2, were compared to assess both models. Both substitutions significantly impair hydrolysis in SUR1 homologs. SUR1_E1506D_ has greater affinity for MgATP than wildtype; SUR1_E1506K_ has reduced affinity. Without Mg^2+^, SUR1_E1506K_ has a greater affinity for ATP^4−^ consistent with electrostatic attraction between ATP^4−^, unshielded by Mg^2+^, and the basic lysine. Further analysis of ND and CHI *ABCC8* mutants in the second transmembrane and nucleotide-binding domains (TMD2 and NBD2) found a relation between their affinities for ATP (±Mg^2+^) and their clinical phenotype. Increased affinity for ATP is associated with ND; decreased affinity with CHI. In contrast, MgADP showed a weaker relationship. Diazoxide, known to reduce insulin release in some CHI cases, potentiates switching of CHI mutants from non-stimulatory to stimulatory states consistent with diazoxide stabilizing a nucleotide-bound conformation. The results emphasize the greater importance of nucleotide binding vs. hydrolysis in the regulation of K_ATP_ channels *in vivo*.

## Introduction

Neuroendocrine ATP-sensitive K^+^ channels, (SUR1/Kir6.2)_4_, couple cell metabolism with K^+^ efflux, and thus membrane potential, in various neurons and endocrine cells including pancreatic islet α-, β-, and δ-cells whose secretory hormones maintain glucose homeostasis. These channels are regulated, in part, by the ATP/ADP ratio. Both nucleotides bind the Kir6.2 pore to reduce the open channel probability (P_o_), while Mg-nucleotide interactions with SUR1 antagonize this effect. The nucleotide effects are modulated further by phosphoinositides, long-chain acyl CoAs, and other factors. In pancreatic β-cells, increased glucose metabolism and the subsequent rise in [ATP/ADP]_C_ reduces K_ATP_ channel activity leading to downstream insulin release. Gain-of-function mutations in the genes that encode either subunit, *ABCC8* (SUR1) or *KCNJ11* (Kir6.2), lead to channel hyperactivity, which attenuates the insulin response to hyperglycemia and causes neonatal diabetes mellitus (ND). Conversely, mutations that reduce channel activity cause congenital hyperinsulinism (CHI) characterized by excess insulin release even during hypoglycemia. ND and CHI mutations perturb regulation of channel activity by at least three distinct mechanisms. Mutations alter either (1) the intrinsic, i.e., ligand-independent, open channel probability, (2) nucleotide inhibition at the Kir pore or (3) Mg-nucleotide-dependent stimulation via SUR1. Additionally, CHI can be due to mutations that disturb trafficking and/or assembly thus reducing the number of functional channels at the plasma membrane (1–3).

SUR1, like other members of the ATP binding cassette family, is reported to have a low level of Mg^2+^-dependent ATPase activity (4, 5). A current, generally accepted model for K_ATP_ channel regulation posits that only post-hydrolytic, MgADP-bound enzymatic intermediates or conformations of SUR1 antagonize nucleotide inhibition at the pore, i.e., that MgATP hydrolysis on SUR1 is required to stimulate channel activity [see Ref. (6) for review]. In line with the current regulatory model, previous studies have argued that SUR1 ND mutations, specifically those that increase Mg-nucleotide-dependent stimulation, increase the SUR1 catalytic rate and/or reduce the off-rate of the MgADP•P_i_ products, thereby augmenting the population of post-hydrolytic stimulatory intermediates (4, 5). A similar argument has been made for the S1369A polymorphism in SUR1, which is reported to increase ATPase activity and thus to increase the risk for type 2 diabetes (7). In contrast to the conventional regulatory model, we have reported that ATP binding, *without hydrolysis*, switches SUR1 from conformations with high affinity for the channel antagonist, glibenclamide (GBC, glyburide in the United States), to conformations with lower affinity for GBC and higher affinity for the channel agonist, diazoxide, i.e., from non-stimulatory to stimulatory states (8, 9). This was demonstrated by removing the Mg^2+^ cofactor required for hydrolysis and by mutating the highly conserved catalytic glutamate, E1506, to glutamine (SUR1_E1506Q_), a substitution that strongly impairs hydrolysis in SUR1 homologs [see, for example, Ref. (10–13)]. Structural studies of ABC proteins indicate that ATP drives dimerization of the cytosolic NBDs upon binding at two sites at the dimer interface. NBD dimerization reconfigures the transmembrane helix bundles (TMD1 and TMD2) from inward-facing to outward-facing orientations [see Ref. (14) for review]. Thus, we proposed that nucleotide-bound (MgATP and/or MgADP) outward-facing conformations of SURs stimulate K_ATP_ channel openings. In support of this updated model, we have also reported that ND mutations distal from the NBDs, SUR1_Q1178R_ and SUR1_R1182Q_, increase affinity for ATP and ADP (9). The current regulatory model predicts that substitutions that impair hydrolysis should impede conformational switching thus leading to underactive channels and CHI. However, a recent report indicates that two catalytic glutamate substitutions, both predicted to impair hydrolysis, lead to opposite disease phenotypes; SUR1_E1506D_ produces hyperactive channels and thus ND while SUR1_E1506K_ forms underactive channels leading to CHI (15). Here, we test the effect of these substitutions on nucleotide affinity by assessing changes in nucleotide-induced conformational switching of SUR1. We found that MgATP affinity correlates with clinical phenotype. The E1506D substitution increases the affinity of SUR1 for MgATP while E1506K reduces affinity. In the absence of Mg^2+^, however, the E1506K substitution *increases* affinity for ATP^4−^, supporting the argument that the Mg^2+^ counterion normally shields the catalytic carboxylate, but is repelled by the substituted lysine. Both substitutions reduce affinity for MgADP, consistent with electrophysiological data indicating that E1506D and E1506K produce channels that are less sensitive to stimulation by MgADP. To support the revised regulatory model and investigate the physiologic relevance of variations in the affinity of SUR1 for nucleotides we tested a larger pool of ND and CHI substitutions in TMD2 and NBD2 and found that ND mutations showed an increase in the affinity of SUR1 for ATP while CHI mutations showed a reduced affinity.

## Materials and Methods

### Reagents

[^3^H]glibenclamide, 5-chloro-*N*-(4-[*N*-(cyclohexylcarbamoyl) sulfamoyl]phenethyl)-2-methoxybenzamide, NET1024250UC at 40–70 ci/mmol was purchased from Perkin-Elmer, Inc., Waltham, MA, USA.

### Plasmids, cells, and membranes

The methods used to generate plasmids, to express wildtype (WT) and mutant SUR1 in *Pichia pastoris* and to isolate membranes were described previously (9) and modified to reduce carryover of endogenous nucleotides, which might affect mutant receptors with highest affinities for nucleotides (8). Amino acid numbering is based on Reference Sequence NM_000352.4. It is worth noting that there are two transcript variants of SUR1/*ABCC8* with lengths of 1581 and 1582 amino acids, respectively. Reference sequences NM_000352.2, NM_000352.3, and NM_000352.4 all specify the 1581 variant referred to as “variant 2” in the NCBI data base. “Variant 1,” with 1582 amino acids, is found in NCBI Reference Sequence: NM_001287174.1. Both variants are expressed in islet cells, with variant 1 being somewhat more frequent (16). It is thus likely that K_ATP_ channels contain a random assortment of both variants. Our unpublished electrophysiological data found no difference between channels of either variant alone (Andrey Babenko and Joseph Bryan). We have used Reference Sequence NM_000352.4 in keeping with most recent reports on *ABCC8* mutations producing ND and/or CHI.

### [^3^H] GBC binding studies

#### Saturation binding assays

Values for K_G_, the dissociation constant for [^3^H]GBC from WT and mutant SUR1 in the absence of added nucleotide, was assessed using saturation binding. Membranes containing WT or mutant SUR1 (~150 pM) were suspended in a Mg^2+^-free physiological salt solution (139 mM NaCl, 5 mM KCl, 1 mM EDTA, 50 mM HEPES, pH 7.4) with increasing concentrations of [^3^H]-labeled GBC. Following incubation for 30 min at 37°C, unbound ligand was removed by rapid filtration as described (17) to determine total bound ligand. Non-specific binding was determined in the presence of 1 μM unlabeled GBC. Specific GBC binding is defined as:

$$\mathrm{Total Bound}=\frac{B_{\mathrm{MAX}}\times G}{K_{G}+G}+\mathrm{Non Specific}$$

Where B_MAX_ is the total amount of receptor, G is the concentration of free [^3^H]-labeled GBC in the reaction, K_G_ is the equilibrium dissociation constant of GBC, and Non-Specific is the amount of non-specific binding typically 10–15% of total bound [^3^H]GBC.

#### Nucleotide inhibition assays

Nucleotide-induced conformational changes in SUR1 were assessed by measuring [^3^H]GBC binding at varying nucleotide concentrations. [^3^H]GBC was held fixed at 1 nM and included the indicated concentrations of nucleotides with or without added Mg^2+^. A creatine phosphokinase-based ATP-regenerating system was used to maintain constant ATP concentrations over a 30 min incubation in experiments with MgATP (free [Mg^2+^] > 1 mM) (18). The stability of ATP levels was verified by luciferase assays (Sigma Chemical Co.). MgADP assays included 10 mM AMP to inhibit endogenous adenylate kinase activity and thus minimize ATP production. The free Mg^2+^ level was 1 mM in experiments with MgADP and MgATP analogs where the ATP-regenerating system was not used. Mg^2+^-free experiments included 1 mM EDTA. Non-specific binding, typically ~10–15% of total bound, was determined in the presence of 1 μM unlabeled GBC. The results are plotted as:

$$\mathrm{Specific Bound GBC}=\frac{\mathrm{Specific Bound(+}X)}{\mathrm{Specific Bound(}-X)}$$

where X is the reagent whose effect is being assayed, e.g., ±ATP^4−^, ±MgATP, etc.

#### Allosteric analysis

**Figure 1:**
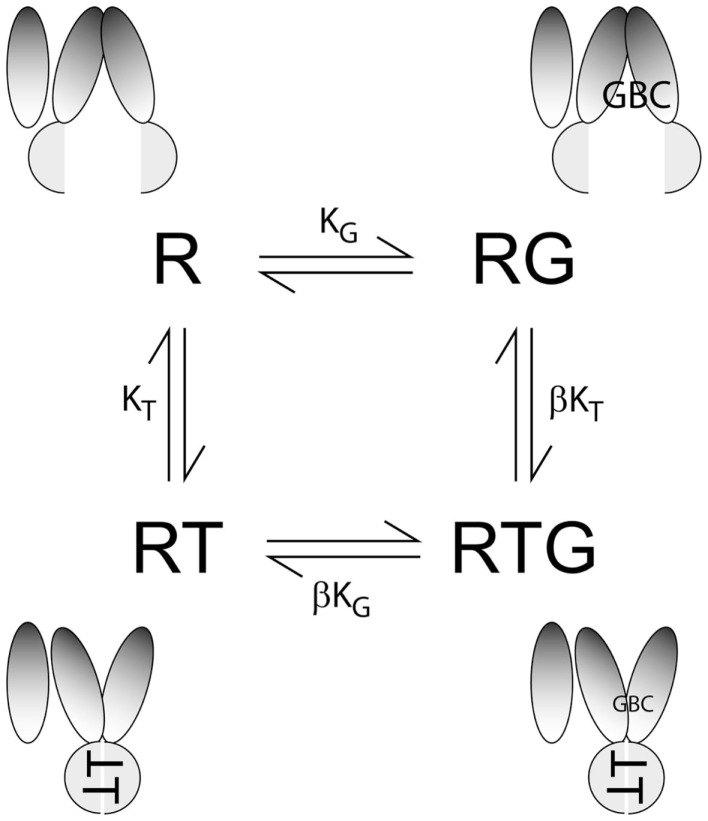
**Four-state equilibrium model**. R, RG, RT, and RTG represent the unliganded receptor, R, and receptors with GBC (G), ATP (T), or GBC + ATP bound, respectively. K_G_ and K_T_ are equilibrium dissociation constants, for GBC and ATP, respectively; β is an allosteric constant; values of β > 0 reflect a reduced affinity of nucleotide-bound SUR for [^3^H]GBC. At given values of K_G_ and K_T_, β specifies the plateau value at a saturating concentration of nucleotide. The cartoons represent the conformations of the four states based on homology models of SUR1 closed and open states derived from crystal structures of evolutionarily related bacterial ABC exporters in the presence (31, 32) and absence of nucleotides (33, 34), respectively.

Adenine nucleotides have a negative allosteric action on the binding of GBC to SUR1 (17, 19, 20). This action is ascribed to nucleotide-dependent switching between a nucleotide-free conformation with highest affinity for [^3^H]GBC and a nucleotide-bound state with reduced affinity (9). We hypothesize that these states are the inward vs. outward-facing conformations of SURs, respectively. A four-state equilibrium model, Figure 1, has been used to analyze the effects of nucleotides on GBC binding. SUR1, like several other ABCC subfamily members, is an asymmetric ABC protein. NBD1, the degenerate site, has a greater affinity for ATP and is presumed to have little or no enzymatic activity, while NBD2 has a lower affinity and ATPase activity. The four-state model presumes NBD1 is nearly saturated at the nucleotide concentrations needed to switch conformations. This is a reasonable assumption for WT SUR1 and the CHI mutants, but may be problematic for ND mutations with higher affinity for ATP.

Derivation of the equations for the four-state equilibrium model followed Wyman and Gill (21) as described (9). The binding equation was obtained from the binding partition function,

$$P=\frac{\left[R\right]+\left[\mathrm{RG}\right]+\left[\mathrm{RT}\right]+\left[\mathrm{RTG}\right]}{R},$$

the sum of the contributions of the different species relative to one reference species, taken here as the unliganded receptor, R. Substituting the dissociation constants gives a binding polynomial:

$$P=1+\frac{\left[G\right]}{K_{G}}+\frac{\left[T\right]}{K_{T}}+\frac{\left[G\right]\left[T\right]}{\beta K_{G}K_{T}}.$$

$\bar{G}$, the amount of [^3^H]GBC specifically bound per mole of SUR1, dependent on both [G] and [ATP], is given by:

$$\bar{G}=\frac{\partial \mathrm{In}P}{\partial \mathrm{In}G}=\frac{\left[G\right]}{P}\frac{\mathrm{\partial P}}{\mathrm{\partial G}}$$

This can be expressed as a binding isotherm:

$$\bar{G}=\frac{G\left(\frac{1}{K_{G}}+\frac{T}{K_{G}K_{T}\beta }\right)}{1+\frac{G}{K_{G}}+\frac{T}{K_{T}}+\frac{\mathrm{GT}}{K_{G}K_{T}\beta }}$$

where K_G_ and K_T_ are the equilibrium dissociation constants for GBC and ATP (T), respectively. β is an allosteric constant; values of β > 0 indicate a reduced affinity of nucleotide-bound SUR for [^3^H]GBC. The experimental data, Specific Bound GBC, are modeled by:

$$\mathrm{Specific Bound GBC}=\frac{\bar{G}}{{\bar{G}}_{\mathrm{T=0}}}.$$

Values for K_T_ and β were estimated by fitting the four-state model to plots of Specific Bound GBC vs. [nucleotide (T)] with fixed K_G_ values that were determined independently using saturation binding assays as described above.

### Statistics

Where indicated the IC_50_ values were estimated by fitting a logistic equation using Origin 2015 (OriginLab Corp., Northampton, MA, USA). The means ±SE are plotted; the number of replicate experiments varies, but *n* ≥ 3 in all cases. ND mutation data are given in red, CHI mutation data are in blue, and WT data are in black throughout. The fitting parameters and SE derived using a four-state model were estimated using *Mathematica* (Wolfram Research Inc., Champaign, IL, USA). Figures were prepared using Origin 2015.

## Results

### Specific mutations have little to no effect on the affinity of inward facing, nucleotide-free conformations of SUR1 for [^3^H]GBC

To assess the effect of the *ABCC8*/SUR1 mutations used in this study on GBC binding the dissociation constants, K_G_, were determined in saturation binding assays in the absence of nucleotides and Mg^2+^. SUR1, like other ABC proteins, is presumably in the inward-facing conformation under this condition. These mutations are not at the putative sulfonylurea binding site [reviewed in Ref. (22, 23)] and have only small effects on GBC binding (see K_G_ values in Table 1).

**Table 1:** **Comparison of binding parameters for neonatal diabetes and congenital hyperinsulinism mutations**.

Mutation	Reference	K_G_	K_T_ (+Mg^2+^)	β	K_T_ (−Mg^2+^)	β	K_D_ (+Mg^2+^)	β
	
		nM	μM		μM		μM	
E1506Q^a^	(8)	0.6 ± 0.2	0.9 ± 0.2	40 ± 20	94 ± 9	40 ± 11	211 ± 34	7.6 ± 2.2
E1506D^a^	(15)	0.4 ± 0.04	3.2 ± 1	8.6 ± 1.5	5570 ± 1200	7.2 ± 1.5	289 ± 122	4.7 ± 2.2
Q1178R^b^	(24)	1.0 ± 0.1	9.2 ± 1.3	10 ± 1	1030 ± 200	9.1 ± 1.7	13.9 ± 2.0	20.7 ± 8.9
I1424V	(24)	0.5 ± 0.03	7.1 ± 2.2	5.6 ± 0.7	2840 ± 700	7.6 ± 1.5	12.1 ± 3.7	14.8 ± 6.5
R1182Q^b^	(24)	0.5 ± 0.15	13.1 ± 2.3	10.3 ± 1.4	11100 ± 1600	4.1 ± 0.4	13.1 ± 2.2	16.4 ± 4.6
WT		0.25 ± 0.02	200 ± 18	13 ± 1	10900 ± 3400	16 ± 11	60 ± 16	14 ± 6.6
S1185A^c^	(9)	0.3 ± 0.05	416 ± 75	4.9 ± 0.5	19100 ± 3600	6.4 ± 1.5	36.6 ± 8	10.4 ± 2.5
C1174F^c^	(9)	0.5 ± 0.04	2690 ± 725	5.9 ± 2.3	>20000	13 ± 6	66 ± 13	7.6 ± 1.7
E1506K	(25)	0.3 ± 0.03	8450 ± 1200	5.5 ± 0.6	256 ± 55	5.3 ± 0.4	>1000	n.d.
G1479R	(26)	0.5 ± 0.04	>10000	n.d.	>20000	n.d.	>1000	n.d.

*^a^Includes data from Ref. (8)*.

*^b^Includes data from Ref. (9)*.

*^c^Personal communication, Dr. Lydia Aguilar-Bryan*.

*ND mutations in red; CHI in blue*.

*K_G_, K_D_, and K_T_ are the dissociation constants for GBC, ADP, and ATP, respectively*.

β is an allosteric constant defined in Section “Materials and Methods.”

*Patients with E1506K (27) and G1479R (26, 28) mutations are responsive to diazoxide*.

#### Two substitutions, E1506D and E1506K, causes of ND and CHI, respectively, have opposite effects on the affinity for MgATP

**Figure 2:**
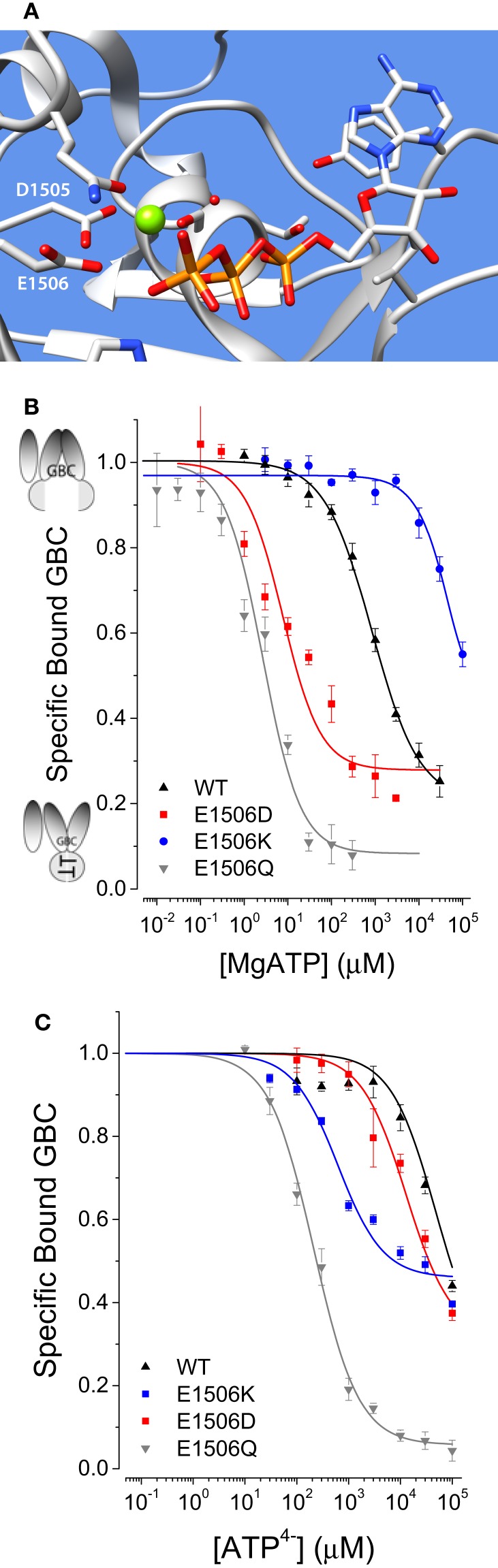
**(A)** Representation of NBD2 based on Sav1866. Comparison of MgATP-induced conformational switching by MgATP **(B)** or ATP^4−^ **(C)**. Here and in subsequent figures ND mutation data are in red, CHI mutation data are in blue, and wildtype (WT) data are in black.

Several ND mutations in SUR1 increase the apparent affinity for ATP (8, 9). To extend these observations two SUR1 substitutions, E1506D and E1506K, well studied at the electrophysiological level (15) and identified with ND and CHI, respectively, were analyzed. E1506 is the essential “catalytic glutamate” in the second nucleotide-binding domain (NBD2) of SUR1 that positions a water molecule for attack on the γ-PO_4_ of ATP (Figure 2A). Substitution of a glutamine (Q) for glutamate (E) at this position results in loss of ATPase activity in other members of the ABC family and has been used to stabilize the nucleotide-bound conformation of other ABC proteins for structural studies. MgATP binding to SUR1_E1506Q_, shown for reference, switches the conformation of SUR1 from a state with high affinity for GBC to one with a ~40-fold reduced affinity. The estimated affinity of SUR1_E1506Q_ for MgATP is several hundred folds greater than WT SUR1 (8). Figure 2B shows that SUR1_E1506D_ also has a greater affinity for MgATP than WT SUR1, while SUR1_E1506K_ has a considerably weaker affinity. The substitution of lysine for glutamate at position 1506 replaces a negative with a positive charge. On simple electrostatic grounds this charge substitution is expected to make the binding of MgATP less favorable when the β,γ phosphates are shielded by Mg^2+^ counterions, which is observed.

Wildtype SUR1 is potentially in a steady-state, slowly hydrolyzing MgATP, while the E1506D and E1506K substitutions are both expected to impair hydrolysis. The reported hydrolytic rates for WT SUR1, under similar conditions, are low and, as described below, the dissociation constant for MgADP is lower than that of MgATP implying product inhibition. Comparison of the approximate EC_50_ values, 50 mM, 900 μM and 10 μM for E1506K, WT and E1506D, respectively, suggests there is an ~5000-fold range in affinities. For comparative purposes, the K_T_ values in Table 1 were estimated from a four-state model assuming the effects of hydrolysis are minimal. This gives a comparable, ~2500-fold difference in affinities for MgATP for SUR1_E1506K_ vs. SUR1_E1506D_, respectively.

#### SUR1_E1506D_ and SUR1_E1506K_ both have increased affinity for ATP^ 4−^

While eliminating Mg^2+^ counterions generally reduces SUR1 affinity for ATP (8, 9), Figure 2C shows that the rank order of the mutations is changed; SUR1_E1506K_ now has a greater affinity for ATP^4−^ than SUR1_E1506D_, the reverse of the result for MgATP. The results imply that without Mg^2+^, electrostatic interactions between lysine and phosphate favor ATP binding to SUR1_E1506K_. The data support the argument that the Mg^2+^ counterion, chelated by the ATP β, γ-phosphates, shields the catalytic glutamate, and that the shorter aspartate and neutral glutamine side chains reduce electrostatic repulsion with the γ-phosphate to improve ATP binding. The results (Table 1) are consistent with the hypothesis that at physiologic concentrations of MgATP SUR1_E1506D_ would spend more time in conformations that stimulate K_ATP_ channel activity vs. WT SUR1, whereas SUR1_E1506K_ would spend less time in stimulatory conformations.

#### SUR1_E1506D_ and SUR1_E1506K_ both have reduced affinity for MgADP

The current regulatory model suggests that ND and CHI mutations would have opposite effects on either the rate of catalysis or the affinity for MgADP. Since both E1506 substitutions are predicted to impair hydrolysis, we tested their effects on MgADP-induced conformational switching. Contrary to the current regulatory model, both E1506 substitutions have reduced affinity for MgADP (Figure 4), consistent with electrophysiological data demonstrating that SUR1_E1506D_/Kir6.2 and SUR1_E1506K_/Kir6.2 channels are less sensitive to MgADP stimulation (15). The neutral E1506Q substitution leads to a similar reduction in MgADP affinity (8).

#### The affinity of SUR1 for ATP correlates with clinical phenotype

**Figure 3:**
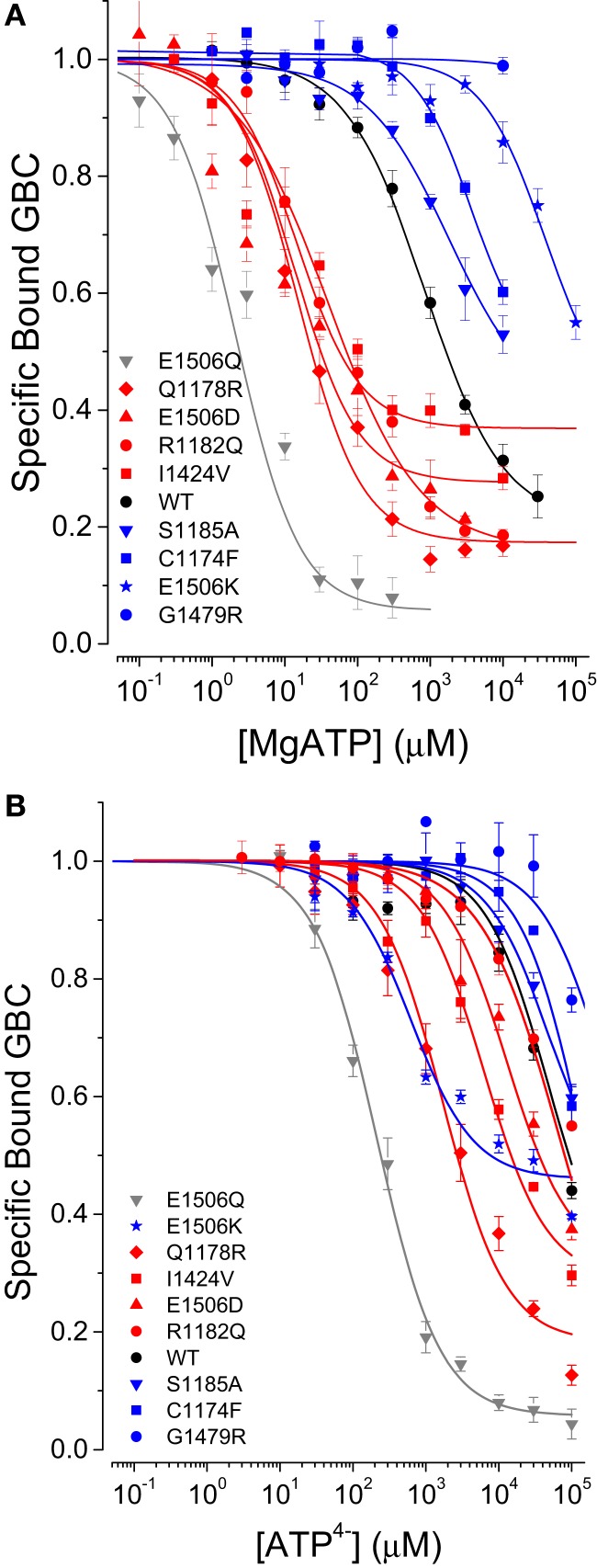
**Comparison of nucleotide-induced conformational switching in WT and SUR1 mutants**. **(A)** MgATP. **(B)** ATP^4−^ + 0.1 mM EDTA.

The results for SUR1_E1506D_ vs. SUR1_E1506K_ and prior data on SUR1 ND mutations (8, 9) suggested a possible correlation between the affinity of a mutant SUR1 for ATP and its clinical phenotype, i.e., ND vs. CHI. To support this hypothesis we analyzed additional mutations including I1424V (ND) and G1479R (CHI) in NBD2 and a cluster of disease causing mutations in TMD2: C1174F (CHI), S1185A (CHI), Q1178R (ND), and R1182Q (ND). We previously showed that SUR1_Q1178R_ and SUR1_R1182Q_, which are on transmembrane helix 15, have an increased affinity for ATP (9). Figure 3A shows that there is a strong correlation of ND (shown in red) and CHI mutations (shown in blue) with their respective clinical phenotype. An increased affinity for MgATP vs. WT SUR1 associates with ND, a decreased affinity with CHI. In other words, the ND mutants switch to a stimulatory conformation, with reduced affinity for glibenclamide, at lower concentrations of nucleotide.

In terms of the conventional model, this correlation could reflect differences in ATPase activity that result, for example, in an increase in the stimulatory MgADP-bound form of SUR1. To assess this possibility directly, we reduced the concentration of Mg^2+^ to submicromolar levels to eliminate ATP hydrolysis and reassessed conformational switching with ATP^4−^. Figure 3B shows that, with the exception of SUR1_E1506K_ discussed above, the ND substitutions all have greater affinities for ATP^4−^ than WT SUR1; the CHI mutations all have reduced affinity. The data for SUR1_E1506Q_ are provided for comparison. For quantitative comparison hydrolysis, in the absence of Mg^2+^, was assumed to be negligible and a four-state equilibrium model was fit to the data. The estimates for K_T_, the ATP dissociation constant and the allosteric constant β are given in Table 1; the curves in Figure 3 were calculated using the four-state model.

#### MgADP affinity is weakly correlated with clinical phenotype

**Figure 4:**
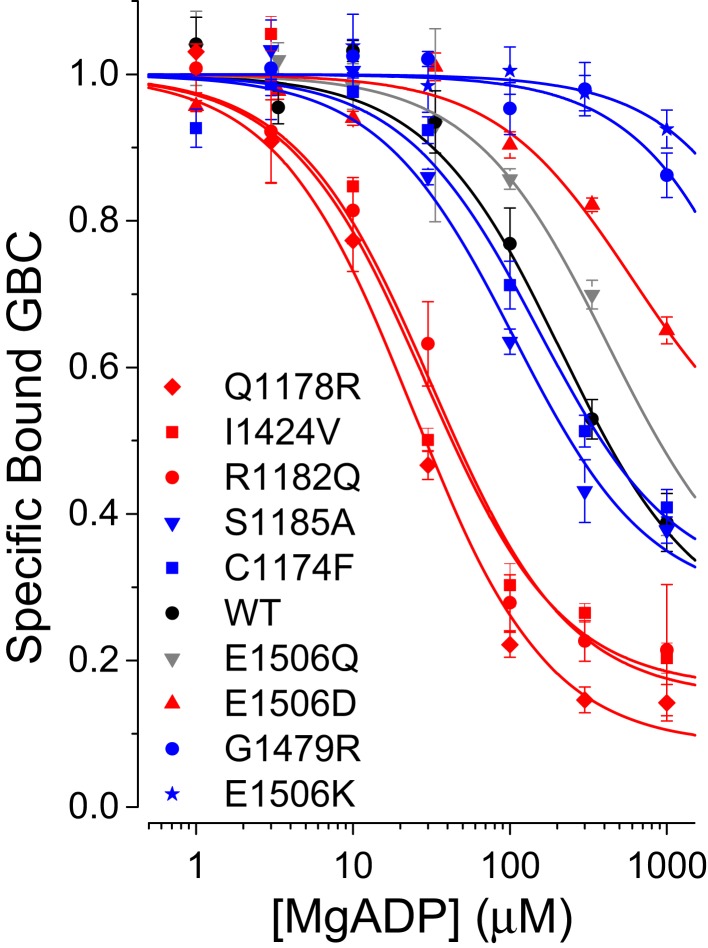
**MgADP-induced conformational switching in WT and SUR1 mutants**.

Based on the conventional regulatory model, in which the post-hydrolytic, MgADP-bound conformation is presumed to be stimulatory, one would anticipate the ND mutants might display greater affinities for MgADP vs. CHI mutants. Figure 4 shows that this is not the case. While three of four ND mutants have a higher affinity for MgADP, SUR1_E1506D_ has a significantly weaker affinity than WT SUR1; two of four CHI mutants have lower affinities for MgADP vs. WT, but the affinities of SUR1_S1185A_ and SUR1_C1174F_ are nearly indistinguishable from WT. It is more difficult, however, to quantify differences in ADP affinity. Membrane preparations have significant endogenous adenylate kinase activity that generates ATP. This activity is partially suppressed by the addition of 10 mM AMP, which alone has no effect on conformational switching. At the higher MgADP levels required to switch conformations of some of the mutant receptors the generated MgATP becomes a significant factor. At 300 μM MgADP, ~4 μM MgATP is generated in our assays (9) and is expected to have significant effects, for example, on SUR1_E1506Q_ and SUR1_E1506D_, which have higher affinities for MgATP, K_T_ values of ~1 and 3 μM, respectively. The results for these mutants suggest MgADP may bind, but not switch conformations and thus may act as a competitive inhibitor. Thus, we have not attempted to compare the actions of MgADP quantitatively.

#### Diazoxide sensitivity of CHI mutants

**Figure 5:**
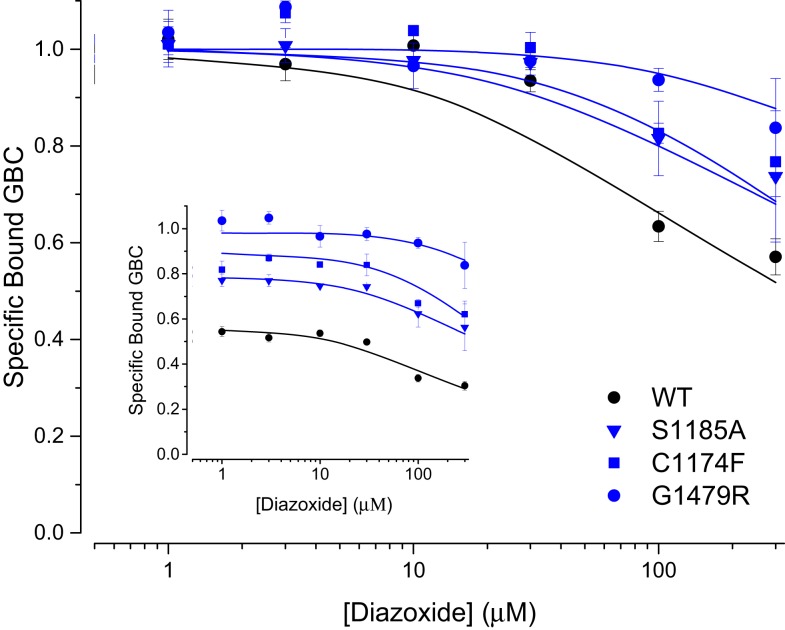
**Diazoxide potentiates conformational switching in WT and CHI SUR1 mutants**. For comparison, the results are normalized to GBC binding in 1 mM MgATP; the data in the inset are normalized to GBC binding in the absence of ATP and show the effect of added nucleotide on each mutant.

Some CHI patients respond to diazoxide therapy and we showed previously that diazoxide, which binds at a site distinct from the NBDs and the GBC binding site, has a positive allosteric effect on MgATP binding to SUR1, i.e., diazoxide interacts preferentially with the nucleotide-bound conformation of SUR1 to stabilize an outward-facing configuration with reduced affinity for GBC (9). We assessed whether diazoxide had a similar action on these CHI mutants by measuring GBC binding at a constant concentration of MgATP (1 mM). Figure 5 shows that diazoxide potentiates the action of 1 mM MgATP on these CHI mutants. The inset shows the relative levels of conformational switching of three CHI mutants by 1 mM MgATP vs. WT SUR1 and indicates the action of diazoxide is effectively the same on each mutant. The result implies these mutations are at residues distinct from the diazoxide binding site and do not significantly affect either the SUR1/diazoxide interaction or the allosteric linkage between the GBC, ATP, and diazoxide binding sites. Patients with the G1479R and E1506K substitutions, as dominant mutations, were responsive to diazoxide (26–29).

## Discussion

Mutations in *ABCC8*/SUR1, the regulatory subunit of K_ATP_ channels, are known causes of ND and CHI. The main finding of this study is the direct correlation between the apparent affinities of mutant SURs for MgATP and their associated clinical phenotype, i.e., ND vs. CHI. With one informative exception, SUR1_E1506K_, the affinities for ATP^4−^ follow the same pattern; a stronger affinity for ATP correlates with ND, weaker affinities correlate with CHI. While we cannot “grade” the clinical phenotype, especially since phenotypes and siblings carrying the same mutation can differ in disease severity, these results establish a relationship between the allosteric properties of SUR1 and clinical phenotype. Knowing how to manipulate these allosteric properties would have clear translational relevance for treating these genetic disorders and type 2 diabetes. The exception to the rule is SUR1_E1506K_, which exhibits the expected reduced affinity for MgATP, the physiologic ligand, but a significantly increased affinity for ATP^4−^. We assume, on simple electrostatic grounds, that without Mg^2+^, the positively charged lysine must attract the phosphates of ATP. The results support earlier studies showing that ATP binding, not hydrolysis, is essential for switching SUR1 from a configuration with highest affinity for sulfonylureas, K_ATP_ channel antagonists, to a “stimulatory” conformation with weaker GBC affinity, but a stronger interaction with diazoxide, a K_ATP_ channel agonist.

The results for MgADP are more complex. Models that assume hydrolysis is required to switch SUR1 to a stimulatory conformation suggest ND mutants would have a greater affinity for MgADP. While this is correct for some ND mutations, e.g., SUR1_Q1178R_, SUR1_E1506D_ has a significantly weaker affinity. As pointed out above, determining affinities for MgADP is difficult as ATP, generated by adenylate kinase, becomes significant at higher ADP concentrations. It is notable that the E1506D, and particularly the E1506Q, substitutions with higher affinities for ATP are not switched appropriately by endogenously generated ATP. A possible interpretation is that MgADP binds, but does not switch these mutant SURs and thus acts as a competitive inhibitor of ATP. It is not clear from the analysis of this limited number of mutants whether this effect is restricted to substitutions for the catalytic glutamate. It is worth emphasizing that based on the relative affinities of WT SUR1 for MgATP vs. MgADP (200 vs. 60 μM) that MgADP generated by hydrolysis could function as a “product inhibitor” to slow turnover.

Early studies (17, 19, 20) on the action of K_ATP_ channel agonists, including diazoxide suggested ATP hydrolysis was required for these compounds to stimulate channel activity. A re-evaluation of these results using both ND and WT SUR1 showed that diazoxide binds preferentially to the ATP-bound form of SUR1 to potentiate ATP-driven receptor switching. The ATP, diazoxide, and GBC binding sites are allosterically linked; added diazoxide effectively increases the affinity of SUR1 for ATP; the ATP-bound conformation of SUR1 has a reduced affinity for GBC.

Some patients with dominant CHI mutations in *ABCC8*/SUR1 are responsive to diazoxide therapy (27, 30). However, usage of diazoxide is limited by low affinity and poor selectivity, resulting in side effects including fluid retention and hypertrichosis. Although CHI SUR1 mutants in this study exhibit reduced sensitivity to switching by stimulatory MgATP, our results indicate that diazoxide potentiates the switching action of MgATP on mutant SURs; specifically, high concentrations of diazoxide (≥100 μM) enhance the switching of millimolar MgATP. The data indicate these CHI mutations neither significantly impair diazoxide binding nor the positive allosteric linkage between diazoxide and MgATP suggesting that more potent, SUR1 selective diazoxide analogs such as NN414 could be more effective at switching SUR1 mutants at lower pharmacological concentrations, thus benefiting CHI patients.

## Conflict of Interest Statement

The authors declare that the research was conducted in the absence of any commercial or financial relationships that could be construed as a potential conflict of interest.
